# Medical Services and Hospitals of the Crown

**Published:** 1914-08-08

**Authors:** 


### MEDICAL SERVICES AND HOSPITALS OF THE CROWN.

### A Survey of Our Organisation.

Now that Great Britain is plunged into the vortex of a war, the like of which has not been seen since Napoleon's time, a brief survey of that part of our medical organisation for military and naval purposes, more especially as it concerns institutions and institutional workers, may be useful. The senior service, the Navy, possesses its own medical service and its own hospitals at Haslar, Chatham, and elsewhere; the famous *Dreadnought* Hospital at Greenwich is a voluntary institution, quite separate from these, but it treats so many sailors that it is certain to be placed freely at the disposal of the Admiralty in case of need. It is only quite lately (The Hospital, July 18, 1914, p. 452) that the shortage of surgeons for the Naval Medical Service was discussed in these columns, and there is no need to repeat here what was then set forth. Suffice it that on August 1 and subsequent days many young doctors were drafted into this service as volunteers to fill the gaps in *personnel*, and that several institutions are short of a resident medical officer in consequence. On Tuesday last a notice actually appeared in the advertisement columns of the *Times* asking for more surgeons for the Navy, eloquent proof of the serious deficiency in numbers, the reasons of which we explained three weeks ago.

Nor need the organisation of the Royal Army Medical Corps of the Regular Army detain us, for it is self-contained and its cadres are full when mobilisation is completed. The only point of contact of this part of the medical service of the Army with the institutional world lies in the fact that a fair percentage of institutional servants—chiefly porters—are Army reservists and have been in many instances summoned away to rejoin the colours.

### The Territorial System.

But with respect to the citizen troops, fashioned six years ago by Lord Haldane out of the old Volunteers, and designated the Territorial Force, the case is different. The organisation of the medical part of this Force concerns very intimately and vitally the hospitals of the kingdom: not only their medical staffs, but also many nurses, are members of this organisation, and many of the most important voluntary hospitals fill integral and official parts of the scheme.

On many occasions during the past six years various aspects of the Territorial Medical Service have been the subject of expert description and comment in these pages. All that can be attempted now is a brief general description of the various parts which make up the whole. The Royal Army Medical Corps (Territorial Force) is divided into several categories, with different duties and special purposes to serve. In the first place there are a large number of Field Ambulances, of which each division of the Force possesses three. A Field Ambulance is technically a unit—that is, it is self-contained and complete within itself. Each Field Ambulance consists of ten officers—nine at least of whom must be medical men—and about 220 rank and file, with a number of ambulance waggons, water-carts, baggage-carts, and all the equipment necessary for setting up a field hospital at a moment's notice whenever required. Moreover, each Field Ambulance is mobile: without assistance from any other troops it can pack up its marquees and tents in a short space of time, take the road, travel to any required destination, and start its field hospital in working order all over again. Each Field Ambulance is commanded by a lieutenant-colonel (a Territorial civilian officer who is as a rule in active medical practice "off parade"), and the junior officers range from majors and captains to lieutenants. Each mounted brigade has an ambulance of its own, organised in the same way except that its scale is rather smaller. There are six officers and rather over a hundred men, and the ambulance waggons are somewhat lighter; otherwise it is a replica of the larger unit.

### Secretaries and the Field Ambulances.

The officers of these units are, as has been said, medical men holding Territorial commissions. The rank and file are drawn from many trades. A certain number of chemists' assistants and travellers for drug firms are enlisted to act as compounders, and some few institutional assistant secretaries and other subordinates of the secretaries' offices carry out clerical duties in the Field Ambulances, and a very important part of the functions of the corps these duties are. But, broadly speaking, the *personnel* of the Field Ambulances are drawn from the same medley of occupations as in the combatant units: clerks, shopkeepers, farmers' sons, and two or three score of other descriptions apply to them.

There are, in addition to the members of these Field Ambulances, other men, whose duty it is to go up to the fighting line. These comprise, first, the Royal Army Medical Corps (T.F.) officers attached to fighting units in medical charge; infantry, yeomanry, artillery, engineers, etc. Second, the stretcher-bearers whom the medical officers are expected to train: these men are not enlisted in the R.A.M.C. at all, but are members of the particular fighting unit in which they act. As a rule, in peace time they are bandsmen, and their due training in stretcher duties is extremely difficult. There are also, nominally at least, selected men of the R.A.M.C. who, in mobilisation, are attached to fighting units to act as water and sanitary police: they are expected to see that the troops conform at least to the rudiments of military hygiene.

So much for the fighting line. The foregoing members of the Territorial Medical Service may, one and all, find themselves under fire at any moment during an engagement. It is their duty to get sufficiently near the troops to succour the wounded promptly; and, although their camps, waggons, and persons are protected from purposeful attack by the enemy, and from capture, they must, to fulfil their mission properly, go into the danger zone to an extent which exposes them to appreciable risks of casualties from hostile fire.

Territorial General Hospitals.

For in the rear of these portions of the medical system are stationed the general hospitals of the Territorial Force. These are officered by twenty or thirty doctors, most of them surgical and medical specialists from the large centres of institutional eminence, who undertake to attend the sick and wounded of the Force in hospitals designated beforehand for this purpose, or improvised in other situations, as the case may be. One general hospital in London, for example, is commanded by a well-known practitioner who some time ago persuaded the Automobile Club Committee to promise their magnificent club house in Pall Mall as an emergency hospital in the event of war. These general hospitals are not expected or intended to go anywhere near the actual seat of war. They are not mobile, nor are they self-contained; and they depend on the exertions of others—not of themselves—for the collection and transport of their patients. The majority of their *personnel* are wholly untrained in even the rudiments of military discipline, and it remains to be seen how far this defect will be counterbalanced by the very high standard of professional efficiency attained by their medical officers, especially those of what is known as the *à la suite* staff (those who join only when a mobilisation is ordered).

How then is the gap between the Field Ambulances and the general hospitals to be bridged? That is to say, how are the sick and wounded Territorials to be conveyed from the battlefield to the hospitals? The Field Ambulances cannot undertake this duty, for their business is to follow the troops wherever they go and to keep in close touch with them. This important function is allotted to the Voluntary Aid Detachments: in other words, to the efforts of the British Red Cross Society, the St. John Ambulance Association, and similar unsubsidised efforts. These bodies have done splendid service in training the large numbers of patriotic men and women who have thrown themselves with ardour into the work. But hampered by their poverty and by the entire absence of all pecuniary help from the Government, many of them—perhaps the majority—are very badly equipped indeed for the trying duties which may fall to them. This grievance, the making of bricks without straw, has on several occasions been ventilated in these columns, but in vain. When the Territorial Force was being organised the Treasury would not provide money enough to equip this part of the medical service, and the deficiency has never been met since. Within the last three or four years millions upon millions have been spent on various more or less well-intentioned schemes for promoting the happiness of the people, but not a sovereign has been forthcoming to provide this essential part of the Territorial organisation with the materials for making itself efficient. If invasion comes, it can hardly be doubted that hundreds, perhaps thousands, of the lives of Territorial soldiers will be sacrificed through inability to get them quickly from the Field Ambulances to the general hospitals; and heavy will be the responsibility of those whose close-fistedness caused this state of things, and of those in high places whose lack of backbone acquiesced in it. This, however, is no time for recriminations. It cannot be doubted that, in whatever breathing space there still may be, the Voluntary Aid Detachments will do their best to acquire the stocks of all sorts which they will want, and it is to be hoped they may succeed in getting them. In *personnel* they consist mainly, but not exclusively, of women. Many of these women are trained hospital nurses, many are merely ladies of some leisure who have tried their best to fit themselves for a national emergency. All honour to them, and may their labours in this hour of struggle and trial be less formidable than those which confront the similar organisations among our foes!

